# Acute Respiratory Tract Infection and Sudden Sensorineural Hearing Loss: A Multinational Cohort Study

**DOI:** 10.3390/diagnostics15121462

**Published:** 2025-06-09

**Authors:** Chien-Hsiang Weng, Jun-Fu Lin, Jing-Jie Wang

**Affiliations:** 1 Department of Family Medicine, Brown University Warren Alpert Medical School, Providence, RI 02903, USA; chien-hsiang_weng@brown.edu; 2 Brown Health Medical Group Primary Care, Brown University Health, Providence, RI 02903, USA; 3 Department of Medical Research, Taichung Veterans General Hospital, Taichung 40705, Taiwan; lin610820012@vghtc.gov.tw; 4 Department of Otolaryngology, Taichung Veterans General Hospital, Taichung 40705, Taiwan; 5 School of Medicine, National Yang Ming Chiao Tung University, Taipei 11221, Taiwan

**Keywords:** sudden sensorineural hearing loss, respiratory tract infections, multinational

## Abstract

**Background/Objectives:** Sudden sensorineural hearing loss (SSNHL) is an acute condition with unclear etiology, commonly hypothesized to be associated with viral infections. Acute respiratory tract infections (RTIs), particularly those of viral origin, have been implicated in SSNHL through proposed mechanisms such as cochlear invasion and immune-mediated damage. However, robust large-scale epidemiological evidence examining this association remains limited. This study aimed to investigate the potential association between acute RTIs and subsequent risk of developing SSNHL across diverse populations. **Methods:** We conducted a multinational retrospective cohort study using data from the TriNetX Global Collaborative Network. Adults diagnosed with acute RTIs between 1 January 2012 and 30 June 2023 were compared to matched controls without RTI exposure. Patients with predisposing conditions for SSNHL were excluded. Propensity score matching (1:1) was performed by age and sex. SSNHL diagnoses within 60 days post index were analyzed using Cox proportional hazards models. Subgroup and sensitivity analyses were conducted by race, sex, and age strata. **Results:** Among 37 million patients analyzed, individuals with acute RTIs had a lower incidence of SSNHL compared to matched controls. Hazard ratios (HRs) for SSNHL were significantly reduced across all racial groups: Whites (HR: 0.572), Blacks (HR: 0.563), and Asians (HR: 0.409). Subgroup analyses revealed stronger inverse associations in males and younger age groups, particularly those aged 18–25 years. **Conclusions:** Contrary to prior assumptions, acute RTIs were associated with a lower incidence of SSNHL in a large, diverse cohort. While the findings raise the possibility of immunological or physiological factors influencing this association, the results should be interpreted with caution due to unmeasured confounding and the observational nature of the study.

## 1. Introduction

Sudden sensorineural hearing loss (SSNHL) is characterized by a rapid onset of hearing loss, typically defined as a decline of at least 30 decibels across three contiguous frequencies within a 72 h period. The annual incidence of SSNHL is estimated to range from 5 to 20 cases per 100,000 individuals, although recent data suggest that incidence rates may be rising due to increased awareness and improved diagnostic capabilities [1,2]. Despite its clinical significance as an otologic emergency, the precise etiology of SSNHL remains elusive. Proposed mechanisms include viral infections, autoimmune responses, vascular compromise, and cochlear membrane ruptures [3]. Immediate intervention with corticosteroids remains the primary treatment modality; however, outcomes are highly variable and depend on factors such as age, initial severity, and time to treatment initiation [4].

Among the hypothesized causes, viral infections have long been considered a key factor. Herpes simplex virus, cytomegalovirus, and influenza viruses are among the agents suspected of direct cochlear invasion or immune-mediated cochlear damage [5]. Acute respiratory tract infections (RTIs), which include both upper and lower respiratory tract illnesses, are among the most common infectious diseases worldwide and are predominantly viral in origin. Pathogens such as influenza, respiratory syncytial virus (RSV), rhinovirus, and coronaviruses are frequent culprits [6]. In addition to pulmonary complications, respiratory viruses have been implicated in extrapulmonary manifestations, including neurological and otologic sequelae. Animal studies and small clinical series have proposed that respiratory viral infections might compromise cochlear homeostasis through mechanisms such as endothelial dysfunction, inflammatory cytokine release, or disruption of the blood–labyrinth barrier [6,7].

Previous investigations into the association between acute RTIs and SSNHL have largely been limited to case reports, small cohort studies, or single-center experiences, often without adequate control for confounding factors. These studies generally suggest an increased risk of SSNHL following viral infections, but their findings are inconsistent, and large-scale epidemiological data are lacking [8]. Furthermore, prior research has rarely addressed potential demographic modifiers, such as age, sex, and race, which could influence SSNHL risk and immune responses to infections.

In recent years, emerging evidence has highlighted the complex interplay between systemic infections, immune regulation, and neurological outcomes [6,9]. For example, post-viral immune dysregulation has been implicated in other sudden-onset neurologic conditions, suggesting that host immune responses, rather than direct viral cytotoxicity, may play a pivotal role. However, whether similar mechanisms underlie SSNHL in the setting of acute RTIs remains unclear. Moreover, recent large-scale healthcare databases offer new opportunities to investigate these associations across diverse populations and healthcare settings.

To address these gaps in the literature, we conducted a large, multinational retrospective cohort study utilizing the TriNetX Global Collaborative Network to evaluate the potential relationship between acute RTIs and the subsequent risk of developing SSNHL. By leveraging extensive real-world data from diverse healthcare organizations, we aimed to provide a more robust and generalizable assessment of this association. A better understanding of this relationship could have important clinical implications for SSNHL prevention, early diagnosis, and targeted therapeutic strategies.

## 2. Materials and Methods

### 2.1. Data Source

This retrospective cohort study utilized data from the TriNetX Global Collaborative Network, a comprehensive, federated health research platform comprising 66 participating healthcare organizations (HCOs) worldwide. Data access occurred on 10 July 2024. TriNetX aggregates de-identified clinical data directly from HCOs’ electronic health records (EHRs) and undergoes rigorous quality control processes, including data completeness, standardization, and validity assessments to ensure accuracy and consistency. While TriNetX maintains high data quality standards, the specific identities of contributing HCOs are not disclosed to investigators. Participating organizations typically include large academic medical centers and integrated health systems that provide a broad range of inpatient, outpatient, emergency, and specialty care services. The global scope and diversity of the network enhance the generalizability of findings across populations and care settings.

### 2.2. Patient and Data Selection

All adult patients (aged 18 years and older) who had at least one clinical encounter recorded within the TriNetX network during the study period were eligible for inclusion. Demographic information, including age, sex, and race, was extracted. Due to computational constraints associated with the large cohort size, analyses were conducted separately within each self-reported racial category: Caucasians (Whites), African Americans or Blacks (Blacks), and Asians (Asians).

To minimize confounding from pre-existing risk factors for sudden hearing loss, patients with prior diagnoses of ear diseases were excluded. Exclusion criteria included ICD-10-CM codes for otitis media (H65.90, H66.90), otosclerosis (H80.9), benign paroxysmal positional vertigo (H81.20), benign neoplasms of cranial nerves (D33.3, D33.2), and tinnitus-related conditions (H93.25). Additionally, patients with metabolic comorbidities known to influence vascular health—including hypertension (I10–I1A), diabetes mellitus (E08–E13), and dyslipidemia (E78)—were excluded to reduce baseline differences (Supplement Table S1).

Given the extensive sample size and platform limitations, multivariable logistic regression modeling was not feasible at the data extraction stage. Therefore, propensity score matching (PSM) based on limited but critical demographic covariates was employed in subsequent analyses.

### 2.3. Acute Respiratory Tract Infections (RTIs)

Patients with documented diagnoses of acute RTIs between 1 January 2012 and 30 June 2023 were identified using ICD-10-CM diagnostic codes indicative of viral and bacterial respiratory infections (B00, B01, B02.21, B05, B20, B25, B26, J01–J06, J09–J18, U07.1, U09.9, Z20.822, Z11.52, Z86.16) (Supplement Table S2). Only diagnoses recorded during ambulatory care visits were included to ensure consistent diagnostic settings and reduce misclassification with hospital-acquired infections. Patients had to be aged 18 years or older at the time of RTI diagnosis to be eligible for inclusion in the exposure group.

A control group was similarly constructed from patients who met the baseline inclusion criteria but had no documented RTI diagnosis during the study period. Index dates for the control group were randomly assigned based on healthcare encounter dates to mirror the temporal distribution of index dates in the RTI group, minimizing potential biases related to healthcare utilization patterns.

### 2.4. Sudden Sensorineural Hearing Loss (SSNHL)

**Figure 1:**
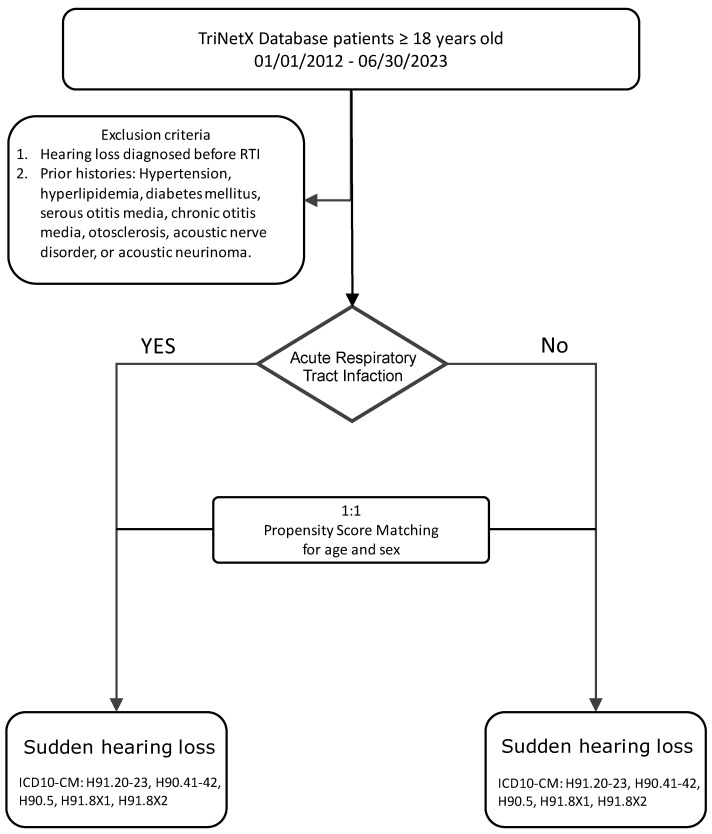
Flowchart.

The primary outcome was the diagnosis of sudden sensorineural hearing loss (SSNHL) within 60 days following the index date (RTI diagnosis or assigned control date). SSNHL events were identified using ICD-10-CM codes H90.4, H90.5, H91.2, H91.8X1, and H91.8X2. Patients with a documented SSNHL diagnosis prior to the index date or within the baseline period were excluded to ensure incident cases only (Figure 1).

The 60-day follow-up window was selected to capture both immediate and subacute SSNHL presentations while limiting potential confounding from unrelated events occurring long after RTI exposure.

### 2.5. Statistical Analyses

To address confounding by baseline demographics, 1:1 propensity score matching without replacement was conducted separately for each racial cohort. Age (continuous) and sex (binary) were used as covariates for matching. A nearest-neighbor matching algorithm with a caliper width of 0.1 standard deviations of the logit of the propensity score was employed to optimize matching precision. The balance between groups post matching was assessed using standardized mean differences (SMDs), with SMDs < 0.1 considered indicative of acceptable covariate balance.

Cox proportional hazards regression models were used to estimate hazard ratios (HRs) and 95% confidence intervals (CIs) for the association between acute RTI exposure and subsequent risk of SSNHL. Time-to-event analyses commenced from the index date and continued until the first SSNHL diagnosis or the end of the 60-day follow-up period. The proportional hazards assumption was tested through Schoenfeld residuals and inspection of log-minus-log plots.

Prespecified subgroup analyses stratified by sex and age category (18–25 years, 26–65 years, ≥66 years) were conducted to assess potential effect modification. Interaction terms were incorporated into Cox models to formally test for heterogeneity across subgroups.

Sensitivity analyses were performed, which included (1) restricting the analysis to patients without prior otologic diagnoses beyond those already excluded, (2) applying alternative PSM strategies with variable caliper widths, and (3) adjusting for healthcare utilization frequency in the sensitivity Cox models.

Statistical significance was determined using two-tailed *p*-values < 0.05. Analyses were conducted using R software (version 4.2.1, R Foundation for Statistical Computing, Vienna, Austria) and SAS software (version 9.4, SAS Institute Inc., Cary, NC, USA).

### 2.6. Ethical Considerations

This study was conducted in compliance with ethical standards for secondary research involving de-identified data. As all information accessed through the TriNetX platform is fully de-identified in accordance with Section §164.514(a) of the HIPAA Privacy Rule, and no protected health information (PHI) was accessed, this study qualifies for exemption from Institutional Review Board (IRB) oversight. The de-identification process was verified by a qualified expert per HIPAA Section §164.514(b)(1), with the latest certification renewed in December 2020. No direct patient contact or intervention was performed. Data outputs were aggregated at the cohort level, preventing re-identification risk. Consequently, informed consent from individual patients was not required, and no additional ethical approval was sought.

## 3. Results

A total of 37,157,115 patients were included for analysis: 29,068,910 Caucasians, 6,083,378 African Americans or Blacks, and 2,004,827 Asians. Before propensity score matching, significant baseline differences were observed between the acute RTI and control groups in all racial cohorts with regard to age and sex (*p* < 0.001 for most comparisons). After 1:1 PSM based on age and sex, balance between groups was achieved, with standardized mean differences below 0.1 for matching variables (Table 1).

The mean age post matching was 26.5 years for Whites, 20.8 years for Blacks, and 23.5 years for Asians. Female patients comprised 57.7% of Whites, 56.5% of Blacks, and 57.4% of Asians in both the RTI and control groups after matching. The average follow-up duration after matching ranged between 22.3 and 25.5 days across subgroups (Table 1).

Across all racial groups, individuals diagnosed with acute RTIs had a significantly lower incidence of SSNHL compared to matched controls. Among Whites, the incidence of SSNHL was 954 cases in the RTI group versus 1534 cases in the control group. The hazard ratio (HR) was 0.572 (95% CI: 0.527–0.620). Among Blacks, 115 cases of SSNHL occurred in the RTI group compared to 186 cases in the control group, with an HR of 0.563 (95% CI: 0.446–0.710). Among Asians, 46 SSNHL cases occurred in the RTI group versus 105 in controls, with an HR of 0.409 (95% CI: 0.289–0.578). All hazard ratios indicated a statistically significant reduction in SSNHL risk associated with acute RTI exposure across racial groups (*p* < 0.001). When expressed as incidence rates, the SSNHL rate among Whites with RTI was approximately 16.4 per 100,000 during the follow-up period, compared to 26.4 per 100,000 in controls. Similar trends were seen among Blacks (RTI group: 8.6 per 100,000 vs. control group: 14.0 per 100,000) and Asians (RTI group: 12.4 per 100,000 vs. control group: 28.2 per 100,000) (Table 2).

Sex-stratified analyses revealed notable differences: Males exhibited a stronger inverse association between RTI and SSNHL than females. In Whites, male patients had an HR of 0.553 (95% CI: 0.484–0.632), compared to 0.738 (95% CI: 0.653–0.835) among females. In Asians, males had an HR of 0.317 (95% CI: 0.165–0.608), while females showed a non-significant HR of 0.643 (95% CI: 0.365–1.132). Among Blacks, male patients had an HR of 0.642 (95% CI: 0.386–1.070), although the confidence interval crossed 1, indicating a lack of statistical significance; for Black females, the HR was 1.284 (95% CI: 0.854–1.929), suggesting no protective effect (Table 2).

These findings suggest that the protective association of RTIs against SSNHL may be more pronounced among males than females, particularly in White and Asian populations. Age-stratified analyses also demonstrated important trends: Younger patients (18–25 years) showed the strongest inverse associations—Whites: HR 0.348 (95% CI: 0.256–0.473); Asians: HR 0.339 (95% CI: 0.108–1.064) (not statistically significant); Blacks: HR 0.894 (95% CI: 0.488–1.636) (not statistically significant). Middle-aged patients (26–65 years) had moderate inverse associations. Older adults (≥66 years) had weaker or non-significant associations—Whites: HR 0.682 (95% CI: 0.561–0.830); Asians: HR 0.449 (95% CI: 0.158–1.276); Blacks: HR 2.460 (95% CI: 0.867–6.984)—suggesting no protective effect and wide uncertainty. Thus, the inverse association between RTI and SSNHL appears strongest among younger individuals, particularly in White patients, and is less evident among older populations.

## 4. Discussion

Our study found that individuals with acute RTIs had a lower incidence of SSNHL across Caucasian, African American, and Asian populations after propensity score matching. The hazard ratio (HR) for SSNHL was 0.623 (95% CI: 0.574–0.675) in Caucasians, 0.619 (95% CI: 0.490–0.781) in African Americans, and 0.439 (95% CI: 0.310–0.620) in Asians, all with statistically significant *p*-values (<0.001). These findings contrast with prior hypotheses suggesting an increased risk of SSNHL following viral infections and highlight potential variations in SSNHL susceptibility across different racial groups.

Sex-based differences also emerged, with males showing a greater difference in SSNHL incidence compared to females in all racial groups. While the difference in Black females was not statistically significant, further studies are necessary to explore potential underlying biological, hormonal, or environmental factors contributing to this trend. Additionally, older individuals (≥66 years) exhibited less variation in SSNHL incidence, with some estimates being non-significant, particularly in Black patients. This pattern may reflect age-related changes in immune function, vascular health, or accumulated comorbidities influencing SSNHL risk. Future investigations should aim to delineate these complex relationships and assess potential mediating mechanisms without assuming direct causality.

The patterns observed indicate differences in SSNHL incidence between individuals with and without RTI. Given prior case reports and smaller studies suggesting a potential association between acute RTI and SSNHL, we initially anticipated a higher incidence of SSNHL among individuals with a recent history of RTI. However, our large-scale multinational analysis showed a consistent reduction in SSNHL risk across racial groups, with variations by sex and age. Younger individuals, particularly those identified as White or Asian, exhibited the greatest difference compared to their matched controls. These findings suggest that demographic factors, immune responses, and possibly genetic predisposition may play a significant role in SSNHL susceptibility. Importantly, our study does not support a positive association between RTI and SSNHL.

### 4.1. Potential Biological Mechanisms

Several biological mechanisms may explain the unexpected inverse relationship observed. One hypothesis involves systemic immune activation during RTI, which could confer transient neuroprotection by modulating inflammatory pathways. Controlled immune responses have been shown to facilitate tissue repair and enhance neuronal resilience [6]. Exposure to respiratory pathogens may stimulate a “trained immunity” phenomenon, wherein immune memory leads to heightened surveillance against unrelated insults, potentially lowering SSNHL risk [10].

The phenomenon of preconditioning—where mild systemic stressors induce protective physiological adaptations—offers another possible explanation. Studies have demonstrated that transient inflammatory stimuli can lead to beneficial changes in gene expression, antioxidant enzyme activity, and cytokine profiles [11]. In the auditory system, experimental models suggest that mild oxidative or inflammatory stress can upregulate endogenous protective mechanisms, influencing susceptibility to noise-induced and ischemic cochlear injuries [12]. Activation of pathways such as nuclear factor erythroid 2–related factor 2 (Nrf2) and heat shock proteins (HSPs) during or after RTIs could contribute to resilience against cochlear insults.

Additionally, upregulation of endogenous corticosteroids during acute infections may play a protective role. Glucocorticoids exert well-documented anti-inflammatory and neuroprotective effects, particularly in mitigating cochlear inflammation and oxidative stress [13]. During acute illnesses, elevated cortisol levels may reduce the likelihood of cochlear dysfunction or inflammatory injury [14].

The concept of viral interference further complicates the relationship. Viral interference refers to the phenomenon whereby infection with one virus suppresses superinfection by other viral pathogens. Some respiratory viruses may competitively inhibit the reactivation or replication of latent neurotropic viruses, such as herpes simplex virus (HSV), which has been implicated in SSNHL [6]. Thus, certain RTIs might indirectly reduce SSNHL risk by preventing the activation of viruses more directly harmful to the cochlea.

### 4.2. Confounding Factors and Unaddressed Variables

While biologically plausible mechanisms exist, several unmeasured factors could influence our results. One notable limitation is the lack of stratification based on RTI severity. The clinical spectrum of RTIs ranges from mild upper respiratory infections to severe lower respiratory tract diseases such as pneumonia or severe COVID-19 [15]. Severe infections can elicit robust inflammatory responses, leading to elevated cytokine levels that may disrupt cochlear homeostasis more profoundly than milder infections, thereby increasing the risk of sensorineural hearing loss [16,17]. Future studies incorporating RTI severity stratification are warranted.

Another unmeasured factor is the impact of treatments for RTIs. Many patients are prescribed antibiotics, antivirals, or corticosteroids. Systemic corticosteroid therapy, frequently used for severe RTIs, could independently lower SSNHL risk through anti-inflammatory mechanisms [18,19]. This possibility highlights the importance of collecting treatment data in future studies to separate infection-related effects from medication-related effects.

The COVID-19 pandemic introduces further complexity. SARS-CoV-2 infections differ pathophysiologically from other RTIs, often involving intense systemic inflammation, endothelial dysfunction, and neurological sequelae [20,21]. Several studies have proposed a potential link between COVID-19 and SSNHL [22,23]. In our study, we did not differentiate COVID-19-related RTIs, and future analyses should separately evaluate this subgroup.

Healthcare-seeking behavior bias must also be considered. RTI patients interacted with the healthcare system more frequently, theoretically increasing the chance of SSNHL diagnosis [24,25]. However, paradoxically, a lower SSNHL incidence was observed among RTI patients, suggesting that surveillance bias does not explain the findings.

Another limitation is the relatively short 60-day follow-up window post RTI. Some SSNHL cases associated with delayed immune processes or viral reactivation may manifest beyond 60 days [26]. Future studies should adopt longer follow-up periods.

We also acknowledge that genetic susceptibility might contribute to variations in SSNHL risk across racial groups. Polymorphisms in immune regulatory genes, viral receptor expression, and inflammatory pathways could influence vulnerability to SSNHL after infection [27,28].

Environmental and occupational factors, such as noise exposure, are additional confounders. Patients with RTIs may have reduced noise exposure during illness recovery, potentially lowering SSNHL risk indirectly [29,30].

To further assess the robustness of our findings to potential unmeasured confounding, we conducted an E-value analysis, a quantitative method for sensitivity analysis in observational research [31]. For example, among White patients, the observed HR for SSNHL associated with acute RTI was 0.572, yielding an E-value of 2.89. Among Black patients, the HR of 0.563 corresponds to an E-value of 2.95. The strongest association was observed in Asian patients, with an HR of 0.409 and an E-value of 4.32. These results indicate that an unmeasured confounder would need to be associated with both RTI exposure and SSNHL by a risk ratio of at least 2.89 to 4.32, above and beyond the measured covariates, to fully explain away the observed protective associations. This suggests that the findings are relatively robust to moderate unmeasured confounding.

### 4.3. Comparison with Previous Studies

Prior smaller studies and case reports have linked viral upper respiratory infections with increased SSNHL risk [32,33]. However, our large, propensity-matched cohort found the opposite association. This aligns with emerging evidence suggesting that mild infections can sometimes modulate immune function protectively, as seen in autoimmune disease epidemiology [34].

### 4.4. Clinical and Research Implications

Our findings have important clinical implications. They suggest that not all respiratory viral exposures increase SSNHL risk and that systemic immune responses may occasionally confer resilience. Future research should explore whether trained immunity or immune preconditioning pathways can be harnessed therapeutically.

Future studies should (1) stratify RTI severity and pathogens; (2) collect detailed data on corticosteroid and antiviral treatments; (3) analyze COVID-19 separately; (4) consider extending follow-up periods; and (5) incorporate genetic and environmental exposure data.

## 5. Conclusions

Our multinational cohort study revealed an unexpected inverse association between acute RTIs and SSNHL, which contrasts with prior assumptions. However, given the limitations of observational data, including potential unmeasured confounding and the lack of pathogen-specific or severity data, these findings should be interpreted as hypothesis-generating rather than conclusive. Further research is needed to validate this association and to explore the underlying mechanisms in more detail.

## Figures and Tables

**Table 1:** Demographics before and after propensity score matching.

Caucasians						
	Pre-matching			Post-matching		
	Acute RTI (*n* = 5,813,268)	Control (*n* = 23,255,642)	*p* value	Acute RTI (*n* = 5,813,268)	Control (*n* = 5,813,268)	*p* value
Age, mean (SD), yr	26.5 (20.1)	36.7 (22.8)	<0.001	26.5 (20.1)	26.5 (20.1)	1
Sex, *n* (%)						
Female	3,353,652 (57.7)	12,974,989 (55.8)	<0.001	3,353,652 (57.7)	3,353,652 (57.7)	1
Male	2,458,206 (42.3)	10,274,623 (44.2)		2,458,206 (42.3)	2,458,206 (42.3)	
**African Americans/Blacks**						
	Pre-matching			Post-matching		
	Acute RTI (*n* = 1,332,041)	Control (*n* = 4,751,337)	*p* value	Acute RTI (*n* = 1,332,041)	Control (*n* = 1,332,041)	*p* value
Age, mean (SD), yr	20.8 (17.8)	31.8 (21.2)	<0.001	20.8 (17.8)	20.8 (17.8)	1
Sex, *n* (%)						
Female	753,018 (56.5)	2,688,417 (56.6)	0.292	753,018 (56.5)	753,018 (56.5)	1
Male	578,492 (43.5)	2,061,225 (43.4)	0.333	578,492 (43.5)	578,492 (43.5)	
**Asians**						
	Pre-matching			Post-matching		
	Acute RTI (*n* = 372,434)	Control (*n* = 1,632,393)	*p* value	Acute RTI (*n* = 372,434)	Control (*n* = 372,434)	*p* value
Age, mean (SD), yr	23.5 (19.0)	32.8 (20.3)	<0.001	23.5 (19.0)	23.5 (19.0)	1
Sex, *n* (%)						
Female	213,674 (57.4)	951,766 (58.3)	<0.001	213,674 (57.4)	213,674 (57.4)	1
Male	158,637 (42.6)	679,859 (41.7)		158,637 (42.6)	158,637 (42.6)	

SD: standard deviation.

**Table 2:** Association between acute RTI and sudden sensorineural hearing loss (SSNHL) in different racial, gender, and age groups.

	Follow-Up Time, Days (SD)		Hearing Loss, *n*		HR (95% CI)
	Case	Control	Case	Control	
***White***					
*Total*	25.1 (10.8)	22.8 (12.3)	954	1534	0.572 (0.527, 0.620)
*Sex*					
*Female*	25.7 (10.2)	23.7 (11.7)	454	573	0.738 (0.653, 0.835)
*Male*	23.8 (11.8)	22.2 (12.6)	340	581	0.553 (0.484, 0.632)
*Age group*					
*18–25*	25.3 (10.6)	22.2 (12.6)	57	145	0.348 (0.256, 0.473)
*26–65*	25.1 (10.7)	23.2 (12.0)	568	767	0.691 (0.620, 0.770)
*≥* *66*	23.3 (11.9)	23.2 (12.0)	169	249	0.682 (0.561, 0.830)
***Asian***					
*Total*	24.2 (11.5)	22.3 (12.5)	46	105	0.409 (0.289, 0.578)
*Sex*					
*Female*	24.4 (11.3)	23.2 (11.8)	20	30	0.643 (0.365, 1.132)
*Male*	22.3 (12.7)	21.4 (12.7)	12	37	0.317 (0.165, 0.608)
*Age group*					
*18–25*	24.1 (11.6)	22.2 (12.4)	≤10	11	0.339 (0.108, 1.064)
*26–65*	23.8 (11.8)	22.6 (12.2)	23	45	0.493 (0.298, 0.814)
*≥* *66*	20.6 (13.3)	22.2 (12.3)	≤10	12	0.449 (0.158, 1.276)
***Black***					
*Total*	25.5 (10.4)	23.0 (12.2)	115	186	0.563 (0.446, 0.710)
*Sex*					
*Female*	25.7 (10.2)	23.8 (11.6)	55	40	1.284 (0.854, 1.929)
*Male*	24.0 (11.6)	22.1 (12.6)	25	36	0.642 (0.386, 1.070)
*Age group*					
*18–25*	25.7 (10.2)	22.9 (12.2)	21	21	0.894 (0.488, 1.636)
*26–65*	25.0 (10.9)	23.0 (12.1)	47	57	0.764 (0.519, 1.124)
*≥* *66*	22.0 (12.6)	22.6 (12.3)	12	≤10	2.460 (0.867, 6.984)

All results were calculated after propensity score matching. HR: hazard ratio; CI: confidence interval; SD: standard deviation.

## Data Availability

The data used in this study were collected from the TriNetX Network on 10 July 2024. The data are de-identified and are available from the corresponding author upon reasonable request.

## Supplementary Materials

The following supporting information can be downloaded at: https://www.mdpi.com/article/10.3390/diagnostics15121462/s1, Table S1: Exclusion criteria for patient and data selection; Table S2: Diagnoses included for acute respiratory tract infection (RTI).
