# Supplementary material for: Acute Respiratory Tract Infection and Sudden Sensorineural Hearing Loss: A Multinational Cohort Study
Source: Diagnostics (Basel). 2025 Jun 9;15(12):1462. doi: 10.3390/diagnostics15121462 (PMC12191787; doi:10.3390/diagnostics15121462)
Supplement: Supplementary file 1 [file diagnostics-15-01462-s001.zip › diagnostics-3637585-supplementary.pdf]

Supplement Table S1: Exclusion criteria for patient and data selection

ICD10-CM codes	
H65.90	Unspecified nonsuppurative otitis media, unspecified ear
H66.90	Otitis media, unspecified, unspecified ear
H80.9	Unspecified otosclerosis
H81.20	Vestibular neuronitis, unspecified ear
D33.3	Benign neoplasm of cranial nerves
D33.2	Benign neoplasm of brain, unspecified
H93.25	Central auditory processing disorder
E08-E13	Diabetes mellitus
E78	Disorders of lipoprotein metabolism and other lipidemias
I10-I1A	Hypertensive diseases

Supplement Table S2: Diagnoses included for acute respiratory tract infection (RTI)

ICD10-CM codes	
B00	Herpesviral [herpes simplex] infections
B01	Varicella [chickenpox]
B02.21	Postherpetic geniculate ganglionitis
B05	Measles
B20	Human immunodeficiency virus [HIV] disease (B20)
B25	Cytomegaloviral disease
B26	Mumps
J01	Acute sinusitis
J02	Acute pharyngitis
J03	Acute tonsillitis
J04	Acute laryngitis and tracheitis
J05	Acute obstructive laryngitis [croup] and epiglottitis
J06	Acute upper respiratory infections of multiple and unspecified sites
J09-J18	Influenza and pneumonia
U07.1	COVID-19
U09.9	Post COVID-19 condition, unspecified
Z20.822	Contact with and (suspected) exposure to COVID-19
Z11.52	Encounter for screening for COVID-19
Z86.16	Personal history of COVID-19
